# Circular RNA PVT1 promotes metastasis via regulating of miR‐526b/FOXC2 signals in OS cells

**DOI:** 10.1111/jcmm.15215

**Published:** 2020-04-05

**Authors:** Ming Yan, Hang Gao, Zhenshan Lv, Ying Liu, Song Zhao, Weiquan Gong, Wei Liu

**Affiliations:** ^1^ Department of Spinal Surgery The First Hospital of Jilin University Changchun P.R.China; ^2^ Department of Bone and Joint Surgery The First Hospital of Jilin University Changchun P.R.China

**Keywords:** circPVT1, FOXC2, metastasis, miR‐526b, osteosarcoma

## Abstract

As a class of covalently closed non‐coding RNAs, circular RNAs (circRNAs) are key regulators in various malignancies including osteosarcoma (OS). In the present study, we found that circular RNA PVT1 (circPVT1) was up‐regulated in OS and correlated with poor prognosis of patients with OS. Functionally, we showed that knockdown of circPVT1 suppressed OS cells metastasis. In addition, we found that (forkhead box C2) FOXC2 was a downstream gene in circPVT1‐mediated metastasis in OS cells. We demonstrated that circPVT1 promoted OS cells metastasis via post‐transcriptionally regulating of FOXC2. Furthermore, we revealed that microRNA 526b (miR‐526b) was a key bridge which connected circPVT1 and FOXC2. We showed that miR‐526b was down‐regulated in OS tissue and cell lines. Through a transwell assay, we found that miR‐526b suppressed OS cells metastasis by targeting of FOXC2. We also showed that miR‐526b targeted circPVT1 via similar mircoRNA response elements (MREs) as it did for FOXC2. Finally, we proved that circPVT1 decoyed miR‐526b to promote FOXC2‐mediated metastasis in OS cells. In brief, our current study demonstrated that circPVT1, functioning as an oncogene, promotes OS cells metastasis via regulation of FOXC2 by acting as a ceRNA of miR‐526b. CircPVT1/miR‐526b/FOXC2 axis might be a novel target in molecular treatment of OS.

## 1 INTRODUCTION

As the most common malignancies of bones in children and young adults, the incidence of osteosarcoma (OS) is about 0.5 cases per 100 000 per year annually.^1^ It is reported that approximately 15%‐20% of patients combined with metastases at diagnosis, mostly in the lungs. The prognosis of patients with lung metastasis appears to be depressing, with about less than 20%‐30% of long‐term survivors.^2^ Although the progress of polychemotherapy and surgical resection significantly improved the total survival rates, the prognosis is still of frustration for most patients with metastatic or recurrent OS.^3^ Therefore, seek out new metastatic molecules and deeper elucidation of their working mechanisms remain great importance for targeting treatment of OS.

Circular RNAs (circRNAs) are a class of RNA transcripts that are thought to arise from non‐canonical splicing of linear pre‐mRNAs.^4^, ^5^ Mounting evidence indicates that circRNAs are comprehensively involved in multiple malignant tumours like bladder carcinoma, hepatocellular carcinoma (HCC), non‐small cell lung cancer (NSCLC), colon cancer, gastric cancer and OS.^6^, ^7^, ^8^, ^9^, ^10^, ^11^, ^12^ Circular RNA PVT1 (circPVT1) is located at chromosome 8q24 and is commonly reported as deriving from one exon of its host gene PVT1.^13^ CircPVT1 flanks two long introns (35 269 bp and 41 466 bp), which harbour many Alu repeats and may facilitate circPVT1 formation.^14^ Qin S found that circPVT1 was up‐regulated in NSCLC tissues and cell lines, and circPVT1 promoted NSCLC progression via acting as a competing endogenous RNA (ceRNA) for miR‐497.^15^ Wang Z reported that circPVT1 was overexpressed and facilitated colorectal cancers (CRC) metastasis via sponging of miR‐145.^16^ Presently, the role of circPVT1 in OS remains unclear.

Forkhead box C2 (FOXC2) is a transcription factor belongs to large family of protein, forkhead box. FOXC2 plays crucial roles in multiple cancers and is often highly expressed in malignancies like breast cancer, gastric cancer, cervical cancer and OS.^17^, ^18^, ^19^, ^20^ Cui YM found that FOXC2 was highly expressed in CRC, and FOXC2 enhanced the invasive abilities of CRC cells in vitro and promoted local invasion and distant metastasis in an orthotopic mouse metastatic model of CRC.^21^ Gozo MC reported that FOXC2 was frequently overexpressed and augmented tumour propagation and metastasis in OS.^22^ Zhu KP found that FOXC2 was up‐regulated in OS, and FOXC2 interacted with lncRNA ENST00000563280 and promoted tumour angiogenesis and epithelial‐to‐mesenchymal transition (EMT) in OS.^23^ Up to present, whether FOXC2 can cowork with any circRNAs keeps obscure.

In the current study, we found that circPVT1 was overexpressed in OS tissues and cell lines. We functionally illustrated that circPVT1 was a key regulator for metastasis in OS cells. We demonstrated that circPVT1 promoted OS cells metastasis via regulation of FOXC2 by working as a ceRNA for miR‐526b. Our finds presented a new molecular axis in targeting treatment of OS.

## 2 MATERIALS AND METHODS

### 2.1 Patients and tissue samples

A total of 48 cases of osteosarcoma tissue samples and paired para‐tumour tissue samples were obtained from patients undergoing resection of tumour at The First Hospital of Jilin University between January 2009 and November 2018. All samples were histopathologically confirmed and obtained with informed consent. No patient received preoperative local or systemic anticancer treatment. Tumour stage was classified according to the guidelines of the 7th Edition of the AJCC (American Joint Committee on Cancer) Cancer Staging Manual of TNM. The study was approved by the Institutional Ethics Committee of The First Hospital of Jilin University.

### 2.2 Cell culture

A human osteoblast cell line hFOB 1.19 was cultured in DMEM/F12 (Gibco). Four human osteosarcoma cell lines MG‐63, U2OS, HOS and 143B were cultured in Dulbecco's modified Eagle's medium (DMEM) (Gibco), and all medium were supplemented with 10% (v/v) foetal bovine serum (FBS, Sigma, St. Louis, MO, USA), 100 IU/mL penicillin and 100 mg/mL streptomycin (Baomanbio). All cell lines were cultured at 37°C in a humidified atmosphere containing 5% CO_2_.

### 2.3 RNase R treatment

The procedure was carried out as previously described.^24^ Two mg of total RNA was incubated for 30 minutes at 37°C with or without 5 U/μg RNase R (Epicentre Technologies), and subsequently purified by a RNeasy MinElute Cleaning Kit (Qiagen, Valencia, CA, USA), then analysed by RT‐PCR.

### 2.4 Quantitative real‐time PCR (qRT‐PCR)

All the procedures were carried out as previously described.^25^ Total RNAs were extracted by using of TRIzol reagent (Invitrogen). Total RNA was reverse transcribed to cDNA, and then qPCR was conducted by using a SYBR Green PCR Kit (Takara) and using GAPDH as an internal control. Expression of miR‐526b was determined by stem‐loop primer SYBR Green quantitative real‐time PCR (RiboBio, Co., Ltd. Guangzhou, China) via applying of U6 as an internal control. Expression of targeted genes was calculated by 2^−ΔΔCt^ method. Specific primers were synthesized by RiboBio as listed in Table S1.

### 2.5 Nucleic acid electrophoresis

The cDNA and gDNA PCR products were investigated using 2% agarose gel electrophoresis with TAE running buffer. DNA was separated by electrophoresis at 120 V for 30 minutes. The DNA marker used was Super DNA Marker (CWBIO). The bands were examined by UV irradiation.

### 2.6 Oligonucleotides and plasmids transfection

Scramble small interfering RNA (siSCR) and specific small interfering RNAs targeted circPVT1 (sicircPVT1‐1 and sicircPVT1‐2), wild and mutant circPVT1 overexpression plasmids containing wild (oecircPVT1‐wt) or mutant (oecircPVT1‐mut) miR‐526b binding sites were synthesized by GenePharma (Shanghai, China). FOXC2 overexpression plasmids (oeFOXC2) and FOXC2 silencing plasmids (siFOXC2) were also synthesized by GenePharma and were used to increase or decrease the expression level of FOXC2. To up‐ and down‐regulation of miR‐526b, miR‐526b mimics, negative control mimic (NC mimic), miR‐526b inhibitors and negative control inhibitors (NC inhibitor) were purchased from RiboBio. All the oligonucleotides and plasmids were transfected into OS cells according to different requirements by using a Lipofectamine 2000 (Invitrogen) according to the manufacturer's protocols. The sequences of small interfering RNAs were listed in Table S1.

### 2.7 In situ hybridizations (*IS*H) assay

The procedures were carried out as previously described.^26^ Fresh OS sections were permeabilized with 0.3% Triton X‐100 for 15 minutes and then incubated in a hybridization solution containing special probes (RiboBio) targeting of circPVT1 or miR‐526b supplemented with 1% blocking solution in a humid chamber at 37°C overnight. The next day, the sections were rinsed with a solution of 0.1% Tween‐20 in 4 × sodium citrate buffer (SSC) for 5 minutes, a solution of 0.1% Tween‐20 in 2 × SSC for 5min and a solution of 0.1% Tween‐20 in 1 × SSC for 5 min at 42°C in dark. Lastly, the sections were triply washed with 1 × PBS for 5 min at room temperature and were counterstained by DAPI. All sections were observed and photographed under a microscope (Leica).

### 2.8 Transwell assay

The procedure was carried out as previously described.^26^ HOS and 143B cells were seeded in a 6‐well plate after designated treatments and incubated for 72 hours. The upper chambers were pre‐coated with (for invasion assay) or without (for migration assay) Matrigel (1:20, BD Biosciences, New Jersey, USA) 2 hours before plating the cells. Cells were cultured with serum‐free media and seeded into the upper chambers at a concentration of 1 × 10^5^/mL. Culture medium supplemented with 10% FBS was placed in the lower chambers. After incubation for 12 h, the invaded or migrated cells were permeabilized by methanol for 20 min at room temperature and stained with 0.1% (w/v) crystal violet in a dark room and then be counted.

### 2.9 Western blot assay

Total proteins were harvested by using radio immunoprecipitation assay (RIPA) lysis buffer (Sigma) and qualified by a BCA detecting kit (Keygen) according to the manufactures’ instructions. Proteins samples were electrophoretically transferred onto PVDF membranes (Millipore, Billerica, MA). Then, the membranes were blocked by Bovine Serum Albumin (Sigma‐Aldrich, St. Louis, MO) and bred with primary antibodies at 4°C overnight. Thereafter, the membranes were incubated with secondary antibodies at room temperature for 1 hour. Bands were visualized by an ECL chemiluminescent detection system (Thermo Fisher Scientific).

### 2.10 Dual‐luciferase reporter assay

The procedure was carried out as previously described.^26^ Reporter plasmids containing wild and mutant sequence of circPVT1 (circPVT1‐luc‐wt and circPVT1‐luc‐mut) or FOXC2 (FOXC2‐luc‐wt and FOXC2‐luc‐mut) were designed and chemically synthesized by GenePharma, respectively. The reporter plasmids were cotransfected with miR‐526b mimics or NC mimic and incubated for 48 hours, individually. Luciferase activity was measured with Dual‐Luciferase Reporter Assay System (Promega) according to the manufacturer's protocol.

### 2.11 RNA‐pull down assay

The procedure was carried out as previously described.^27^ In brief, cells were quantitated and treated with 1 mL of cell lysis buffer for 72 hours. Then, cells were rotated overnight at 4°C after adding 1.5 μL of RNase inhibitor, 10 μL of streptavidin agarose beads and 500 pmol/L antisense oligos. Beads were washed 5 times by cell lysis buffer. Total RNAs were subjected to qRT‐PCR analysis.

### 2.12 Statistical analysis

All data were collected from three independent repeated experiments and were expressed as mean ± SD. Statistical analysis was evaluated by using a GraphPad Prism V5.0 (GraphPad Software Inc.) software and SPSS 19.0 statistical software (IBM). Overall survival (OS) was evaluated by Kaplan‐Meier survival curves and compared by log‐rank test. Pearson's chi‐squared test was used to analyse the correlation between circPVT1 and clinicopathological features of patients with OS. Differences between two groups were analysed by the Student's *t*‐test. One‐way analysis of variance was used for analysing of differences among multiple sets of data. Differences were considered significant if *P < .05, **P < .01, ***P < .0001, respectively.

## 3 RESULTS

### 3.1 CircPVT1 is up‐regulated in OS and correlated with poor outcomes

**FIGURE 1:**
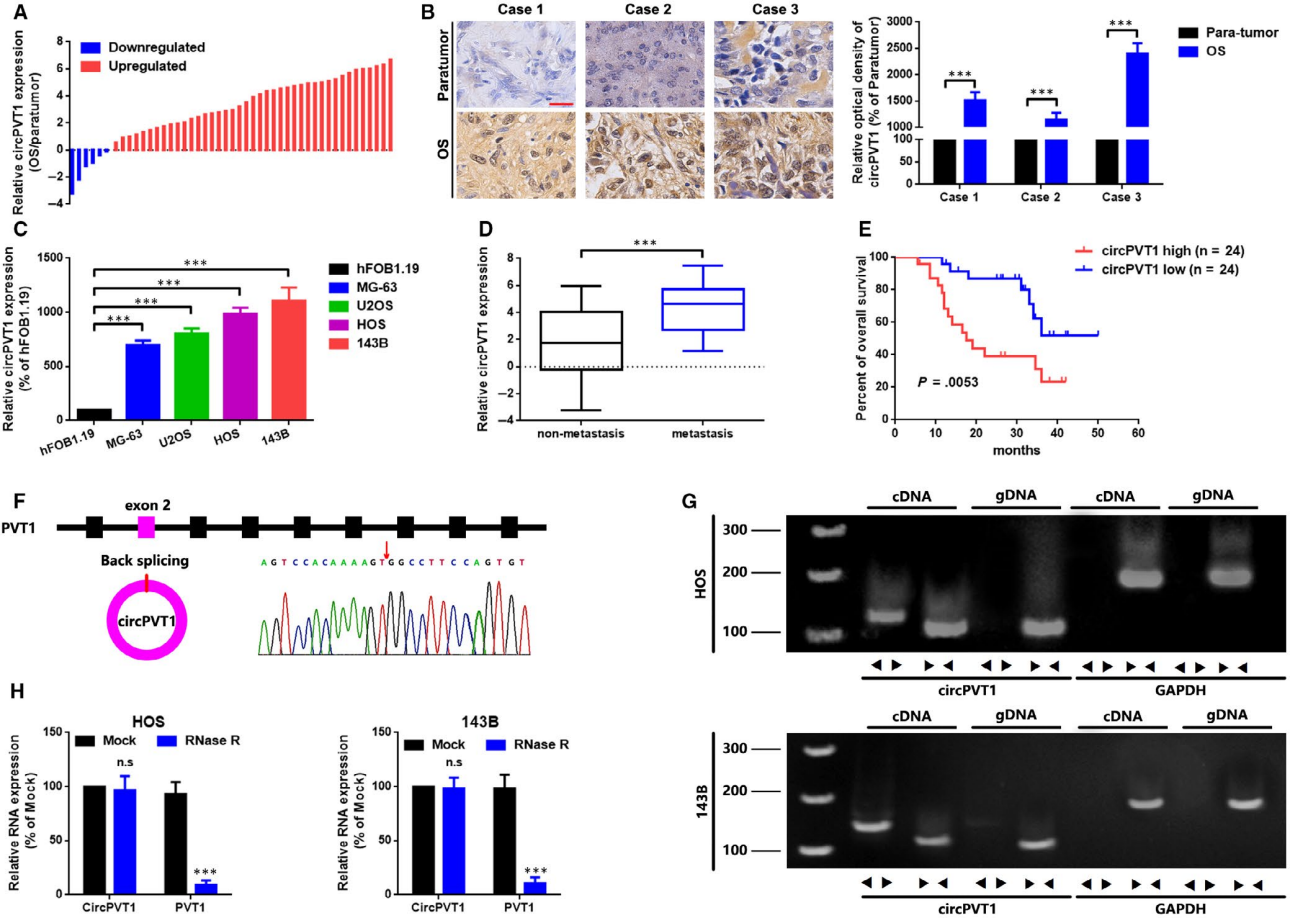
CircPVT1 is up‐regulated in OS and correlated with poor outcomes. A, CircPVT1 expression in 48 paired OS tissue samples was qualified by using a qRT‐PCR analysis via applying of log2 (2^−△△Ct^) method. B, CircPVT1 expression in OS tissue samples and paired para‐tumour tissue samples was determined by an *IS*H assay. Scale bar, 50 µm; magnification, 40×. C, qRT‐PCR was used to measure the expression of circPVT1 in 4 OS cell lines MG‐63, U2OS, HOS and 143B and in a normal human osteoblast cell line hFOB1.19. D, Expression of circPVT1 was higher in patients with lymph node metastasis (N1 and N2, than that in patients without lymph node metastasis (N0). E, Association of circPVT1 expression with overall survival analysis of 48 OS patients, detected by a Kaplan‐Meier analysis, *P* = .0053. F, Sanger sequencing was used to verify the head‐to‐tail splicing of circPVT1. G, RT‐PCR validated the existence of circPVT1 in HOS and 143B cell lines. CircPVT1 in cDNA, instead of genomic DNA, was amplified by divergent primers. GAPDH was used as a negative control. H, Expression of circPVT1 and PVT1 in HOS and 143B cells were determined by RT‐PCR or qRT‐PCR with or without the effect of RNase R. Data were shown as mean ± SD from three independent experiments. ***P < .001 compared to controls

We performed qRT‐PCR to measure the expression of circPVT1 in collected 48 OS tissue samples. As the data presented in Figure 1A, circPVT1 was up‐regulated in most (42/48, 87.50%) OS tissue samples. In addition, we measured the expression of circPVT1 in 3 fresh OS tissue samples by an *IS*H assay. As the representative photos displayed in Figure 1B, circPVT1 was significantly highly expressed in OS tissue samples than that of para‐tumour tissue samples. Further, we checked expression of circPVT1 at cellular level. As the findings showed in Figure 1C, compared to a normal human osteoblast cell line hFOB1.19, circPVT1 was overexpressed in four osteosarcoma cell lines MG‐63, U2OS, HOS and 143B. Even more, we found that up‐regulated circPVT1 was more commonly presented in OS tissue samples with lung metastasis (Figure 1D). Meanwhile, we evaluated the clinical value of circPVT1 in patients with OS. As shown in Figure 1E and Table 1, up‐regulated circPVT1 was closely correlated with advanced clinical stage (*P* = .008), distant metastasis (*P* = .009) and shorter survival rate (*P = *.0053, Figure 1E). Even more, we presented that circPVT1 was derived from exon 2 of PVT1 oncogene, and the head‐to‐tail splicing was confirmed via Sanger sequencing (Figure 1F). Even further, cDNA and gDNA were extracted separately from HOS and 143B cells and subjected to nucleic acid electrophoresis detection. As the results displayed in Figure 1G, circPVT1 could be detected in only cDNA, as no products were detected in the extracted gDNA. Finally, by an RNase R assay, we verified that circPVT1 was more stable than PVT1 after an RNase R treatment (Figure 1H).

**TABLE 1:** Association of circPVT1 expression with clinicopathological features of osteosarcoma

Features	No. of cases	CircPVT1		*P*‐value^a^
		High	Low	
Age at diagnosis				.745
＜18	35	18	17	
≥18	13	6	7	
Gender				.771
Female	27	13	14	
Male	21	11	10	
Histological subtype				.849
Osteoblastic	9	6	8	
Chondroblastic	18	5	6	
Fibroblastic	38	7	5	
Mixed	30	6	5	
Clinical stage				.008
I+IIA	19	5	14	
IIB/III	29	19	10	
Distant metastasis				.009
Absent	21	6	15	
Present	27	18	9	
Tumour size (cm)				.149
<5	39	10	11	
≥5	56	14	13	
Anatomic location				.505
Tibia/femur	36	19	17	
Elsewhere	12	5	7	

^a^ *P*‐value obtained from Pearson Chi‐Square test.

### 3.2 Down‐regulation of circPVT1 suppressed metastasis in HOS and 143B cells

**FIGURE 2:**
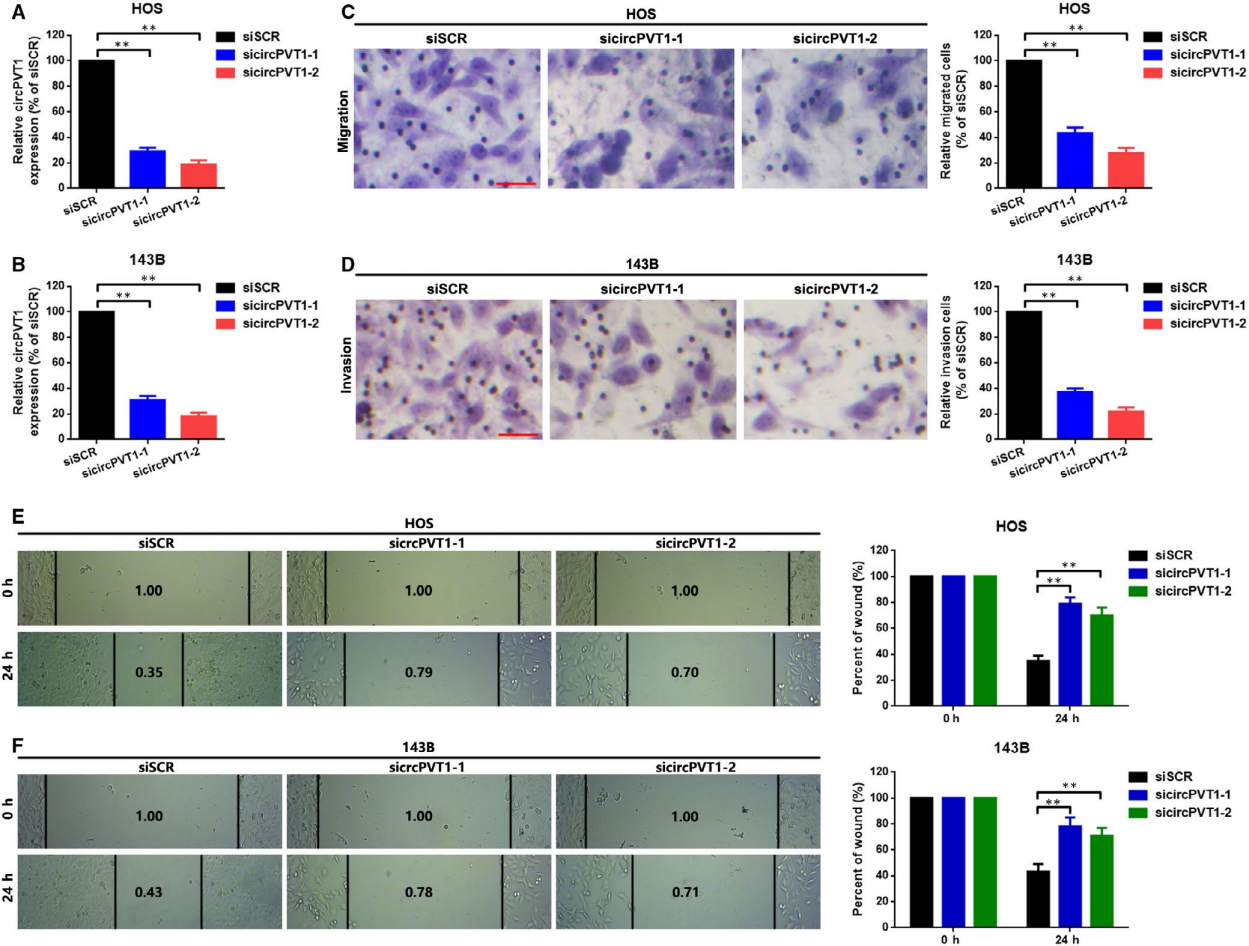
Down‐regulation of circPVT1 suppressed metastasis in HOS and 143B cells. A and B, CircPVT1 was knocked down by specific siRNAs sicircPVT1‐1 and sicircPVT1‐2 as confirmed by a qRT‐PCR assay. C and D, Transwell assays were performed after knocking down of circPVT1 in HOS and 143B cells. The migrated or invaded cells were counted in 10 randomly chosen microscopic fields (200×) of each experiment and pooled. E and F, Migration ability of HOS and 143B cells were checked by a wound healing assay. Data were shown as mean ± SD from three independent experiments. **P < .01 as normalizing and comparing with siSCR group

To explore whether circPVT1 play a role in OS cells metastasis, we constructed in vitro loss of function assays. We transfected circPVT1 specific siRNAs (sicircPVT1‐1 and sicircPVT1‐2) into HOS and 143B cells, and the expression of circPVT1 as well as lncRNA PVT1 was measured by a qRT‐PCR assay. As the outcomes displayed in Figure 2A, B and Figure S1A, B, compared to a scramble control (siSCR), circPVT1 was significantly knocked down by sicircPVT1‐1 and sicircPVT1‐2. However, the changes of lncRNA were not significant. Further, a transwell assay (Figure 2C, D) and a wound healing assay (Figure 2E, F) indicated that down‐regulation of circPVT1 suppressed migration and invasion in HOS and 143B cells.

### 3.3 CircPVT1 promoted metastasis via up‐regulation of FOXC2 in HOS and 143B cells

**FIGURE 3:**
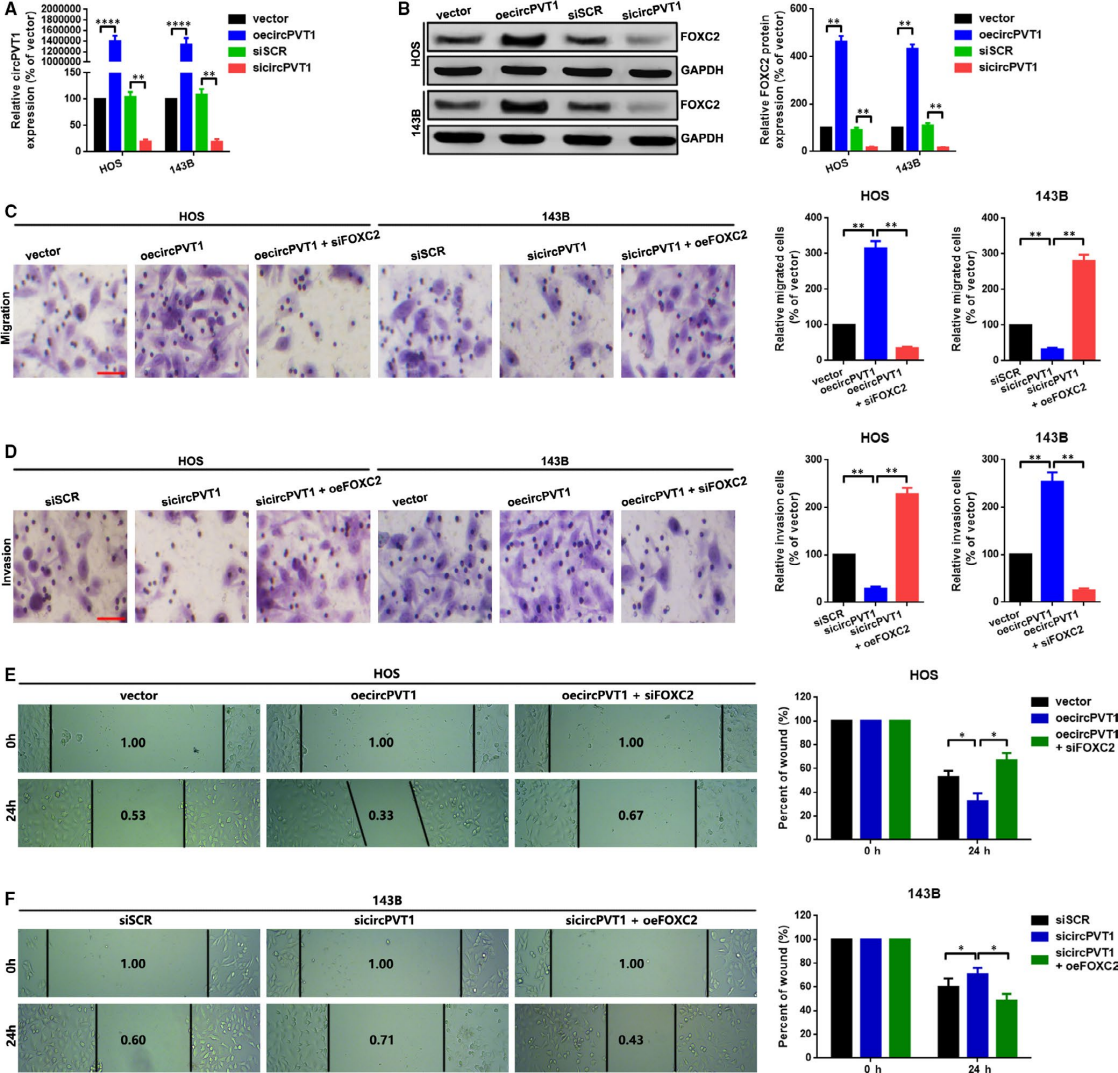
CircPVT1 promoted metastasis via up‐regulation of FOXC2 in HOS and 143B cells. A, CircPVT1 was overexpressed and knocked down by transfection of oecircPVT1 and sicircPVT1 as measured by a qRT‐PCR, respectively. B, Expression of FOXC2 after up‐ and down‐regulation of circPVT1 was measured by a Western blot. C, A transwell migration assay was performed in HOS cells with three groups (vector, oecircPVT1 and oecircPVT1+siFOXC2), and in 143B cells with another three groups (siSCR, sicircPVT1 and sicircPVT1+oeFOXC2) D, A transwell invasion assay was also applied in HOS cells with three groups (siSCR, sicircPVT1 and sicircPVT1+oeFOXC2), and in 143B cells with another three groups (vector, oecircPVT1 and oecircPVT1+siFOXC2). The migrated or invaded cells were counted in 10 randomly chosen microscopic fields (100×) of each experiment and pooled. E and F, Migration ability of HOS (E) and 143B (F) cells was determined by a wound healing assay. Each sample was run in triplicate and in multiple experiments for mean ± SD. **P < .01 compared to controls, respectively

FOXC2 is a key regulator in several cancers metastasis including OS.^21^, ^22^, ^28^, ^29^ We attempted to investigate the relationship between circPVT1 and FOXC2. We firstly constructed cell models with different circPVT1 expression level in HOS and 143B cells via transfection of a circPVT1 overexpression plasmid (oecircPVT1) and a specific circPVT1 siRNA, individually. As the data presented in Figure 3A, circPVT1 was significantly overexpressed and knockdown in OS cells, correspondingly. We then determined the effect of circPVT1 working on FOXC2. As the result of Western blot displayed in Figure 3B, FOXC2 is positively regulated by circPVT1. Functionally, as the representative figures of transwell assay displayed in Figure 3C, D, we found that up‐regulation of circPVT1 promoted OS cells migration and invasion, and the facilitative effect was abolished by a suppression of FOXC2, and vice versa. Also, a similar tendency was displayed by using of a wound healing assay (Figure 3E, F).

### 3.4 CircPVT1 regulated FOXC2 partially via miR‐526b

**FIGURE 4:**
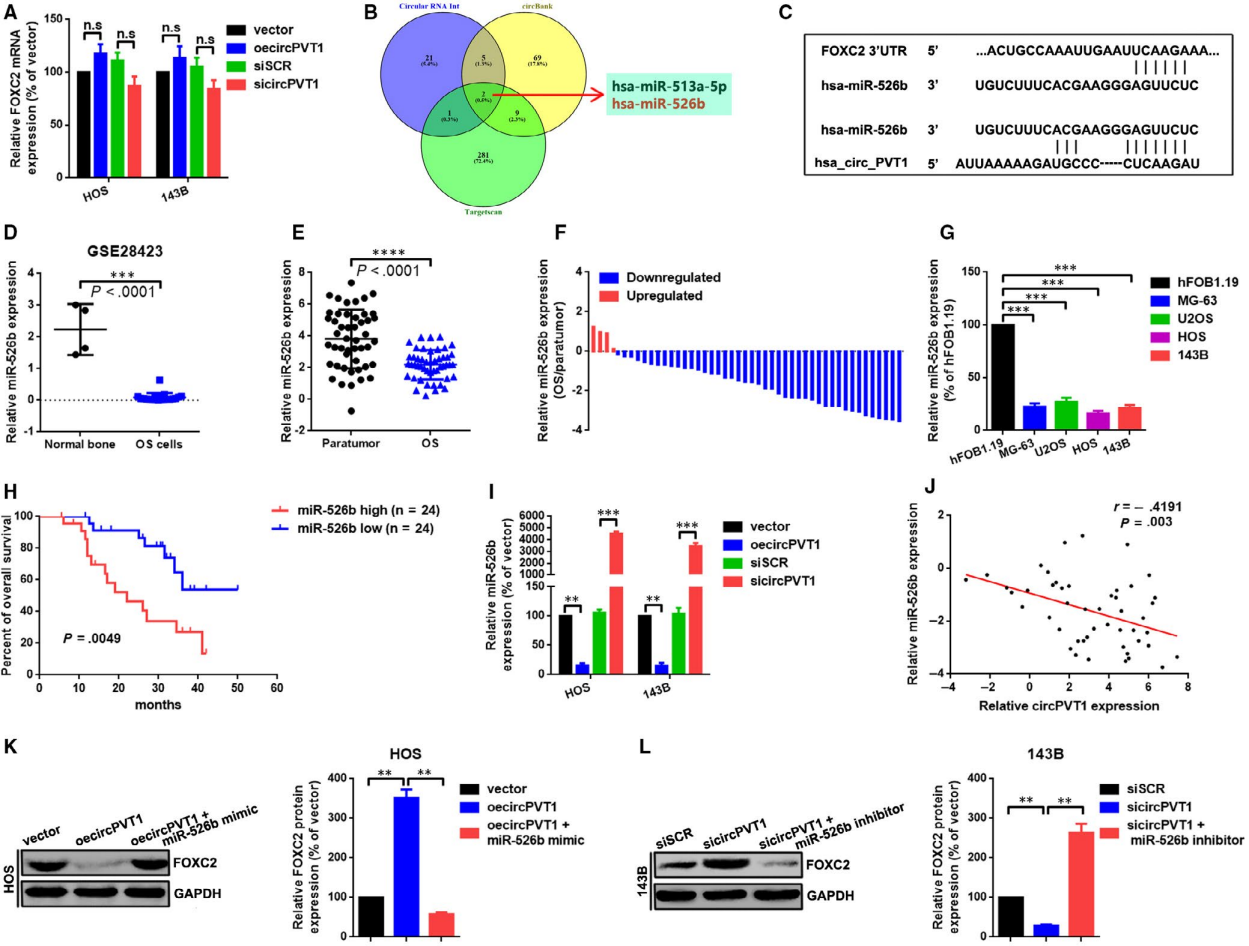
CircPVT1 regulated FOXC2 partially via miR‐526b. A, Expression of FOXC2 mRNA after up‐ and down‐regulation of circPVT1 was evaluated by a qRT‐PCR assay. B, MiRNAs targeted circPVT1 and FOXC2 were predicted by using of circBank, circular RNA interactome and Targetscan. MiR‐526b and miR‐513a‐5p were overlapped in the aforementioned 3 databases. C, A diagram presented the seeding sequences of miR‐526b in circPVT1 and FOXC2. D, Expression of miR‐526b was significantly lower in OS than that of in normal bone according to an analysis of GEO database GSE28423. E and F, Expression of miR‐526 in collected 48 OS samples was measured by a qRT‐PCR assay. (g) Expression of miR‐526b at cellular level was determined by a qRT‐PCR assay. H, A Kaplan‐Meier analysis indicated that lower miR‐526b was correlated with shorter overall survival in 48 OS patients. I, Expression of miR‐526b after up‐ and down‐regulation of circPVT1 was determined by a qRT‐PCR assay, either. J, Correlation between circPVT1 and miR‐526b as measured by Pearson's correlation coefficient. K and L, Expression level of FOXC2 protein after cotransfection of oecircPVT1 and miR‐526b mimics (K) or sicircPVT1 and miR‐526b inhibitor (L) was measured by a Western blot. Each sample was run in triplicate and in multiple experiments for mean ± SD. ^n.s^ *P* > .05, **P < .01 and ***P < .001 compared to controls, individually

We have showed that circPVT1 regulated FOXC2‐mediated metastasis in OS cells. We then tried to explore how circPVT1 regulate FOXC2. We found that the mRNA levels of FOXC2 only changes a little after up‐ and down‐regulation of circPVT1 (Figure 4A). This phenomenon indicated that some non‐coding RNAs might involve in this process at a post‐transcriptional level. It is well known that circular RNAs regulate their downstream genes via a mechanism of ceRNA post‐transcriptionally. We used 3 online prediction software circBank (http://www.circbank.cn/index.html), circular RNA interactome^30^ and Targetscan (http://www.targetscan.org/vert_72/) to infiltrate potential miRNAs that might interacted with circPVT1 and FOXC2. 2 miRNAs (miR‐526b and miR‐513a‐5p) were overlapped in the aforementioned 3 databases (Figure 4B and 4C). We selected miR‐526b for a further study as it was down‐regulated in OS through analysing of GEO datasets GSE28423 (Figure 4D ; Figure S2A, B). In addition, we found that miR‐526b was down‐regulated and was closely correlated with poor prognosis in patients with OS (Figure 4E, F). A further cellular level of detection also indicated a similar result (Figure 4G). Even more, we showed that low expression of miR‐526b was closely correlated with shorter overall survival of patients with OS (Figure 4H). Subsequently, we demonstrated that up‐ and down‐regulation of circPVT1 negatively regulated miR‐526b expression (Figure 4I). Meanwhile, we illustrated that there was an inverse correlation (*r* = −.5141, *P* = .0002) between circPVT1 and miR‐526b in collected 48 OS tissue samples (Figure 4J). Finally, we illustrated that the facilitative effect of oecircPVT1 on FOXC2 protein was abolished by up‐regulation of miR‐526 (transfection of miR‐526 mimic), and vice versa (Figure 4K, L).

### 3.5 MiR‐526b suppressed metastasis via directly targeting of FOXC2 in HOS and 143B cells

**FIGURE 5:**
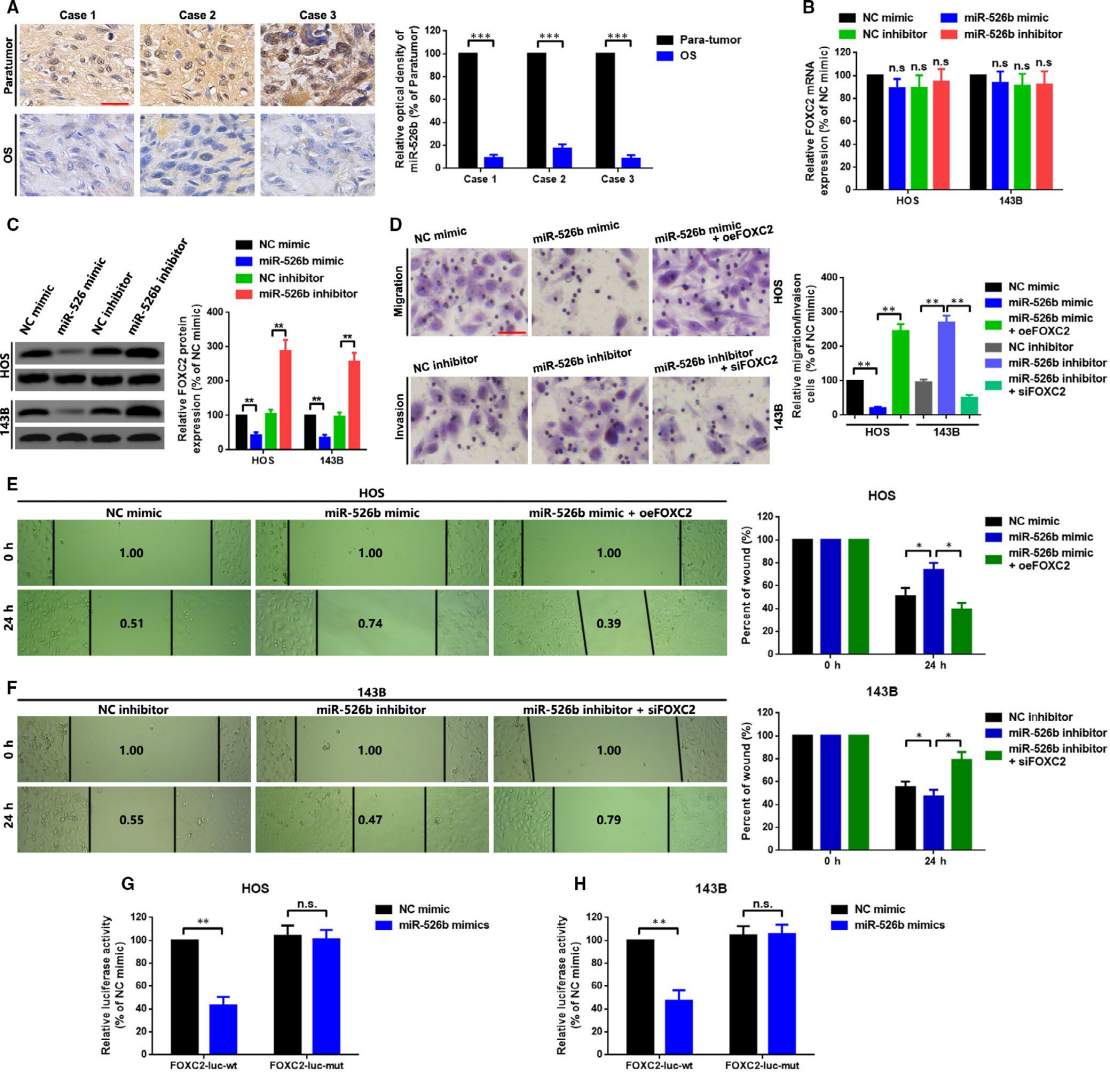
MiR‐526b suppressed metastasis via directly targeting of FOXC2 in HOS and 143B cells. A, MiR‐526b expression in OS tissue samples was qualified by using an *IS*H assay. B and C, Expression of FOXC2 was determined by a qRT‐PCR assay (B) and a Western blot assay (C) after up‐ and down‐regulation of miR‐526b. D, A transwell migration assay was performed in HOS cells with three groups (NC mimic, miR‐526b mimic and miR‐526b mimic+oeFOXC2), and a transwell invasion assay was performed in 143B cells with another three groups (NC inhibitor, miR‐526b inhibitor and miR‐526b inhibitor+siFOXC2). E and F, Migration ability changes of HOS (E) and 143B (F) cells was checked by a wound healing assay. G and H, Cotransfection of wild or mutant reporter plasmids (FOXC2‐luc‐wt or FOXC2‐luc‐mut) and miR‐526b mimic in HOS (G) and 143B (H) cells, separately. Luciferase reporter assay was applied to detect the luciferase activities. Each sample was run in triplicate and in multiple experiments for mean ± SD. ^n.s^ *P* > .05, *P < .05, **P < .01 and ***P < .001 compared to controls, respectively

In this section, we further illustrated the role of miR‐526b in OS cells metastasis. We firstly revealed that miR‐526b was down‐regulated in OS tissue samples by an *I*SH assay (Figure 5A). We then demonstrated that up‐ and down‐regulation of miR‐526b negatively regulated FOXC2 protein but not mRNA expression (Figure 5B, C). Functionally, we found that up‐regulation of miR‐526b suppressed OS cells migration and invasion, and the suppressive effect was reversed by an overexpression of FOXC2 (miR‐526b mimic + oeFOXC2). By contrast, down‐regulation of miR‐526b promoted OS cells migration and invasion, and the facilitative effect was abolished by a knockdown of FOXC2 (miR‐526b inhibitor+siFOXC2) (Figure 5D). Even more, a similar tendency was found by a wound healing assay (Figure 5E, F). Lastly, through a luciferase assay, we showed that FOXC2 was a direct target of miR‐526b (Figure 5G, H).

### 3.6 CircPVT1 decoyed miR‐526b to promote FOXC2‐mediated metastasis in HOS and 143B cells

**FIGURE 6:**
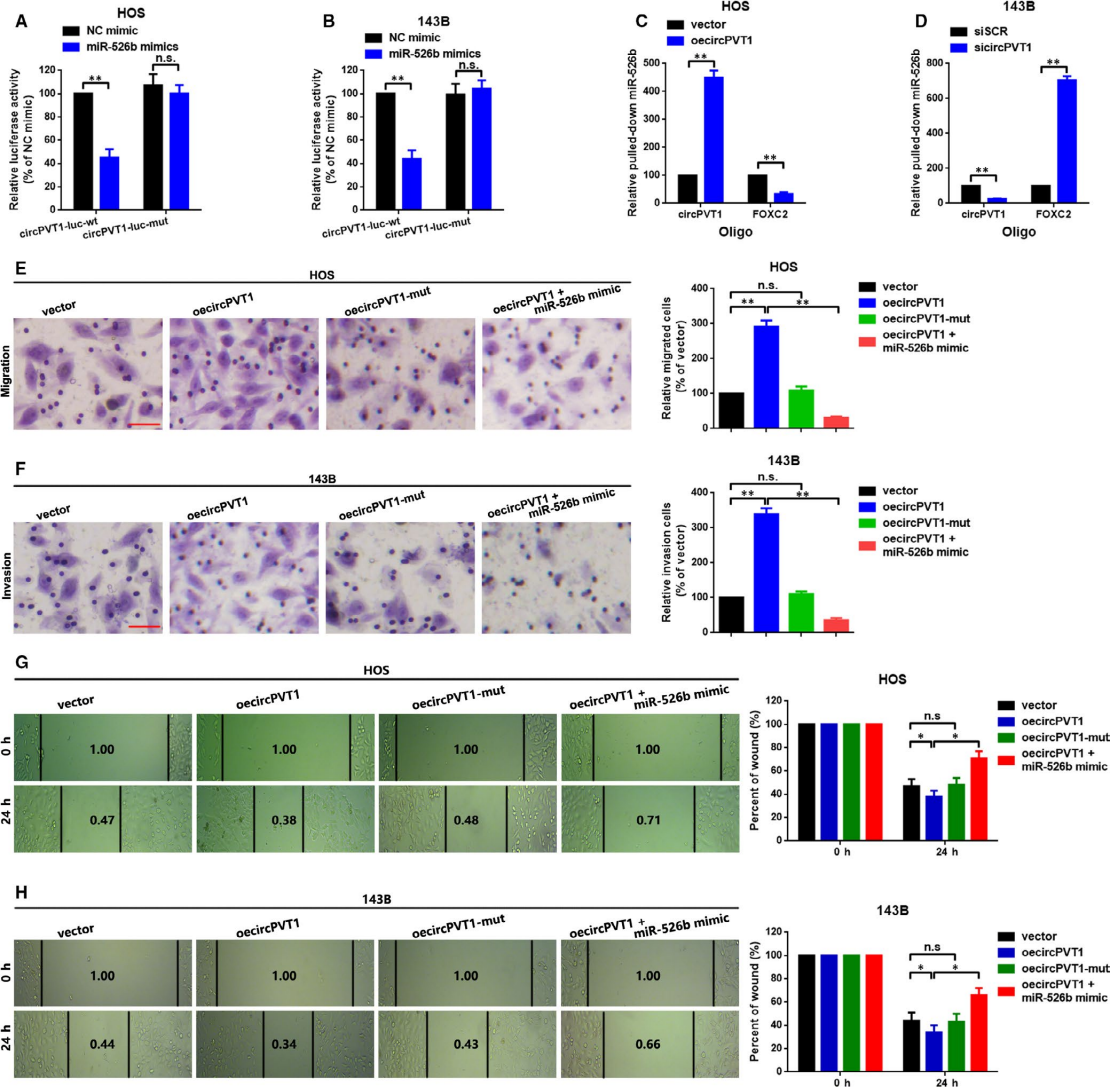
CircPVT1 decoyed miR‐526b to promote FOXC2‐mediated metastasis in HOS and 143B cells. A and B, Wild or mutant reporter plasmids (circPVT1‐luc‐wt or circPVT1‐luc‐mut) and miR‐526b mimic were cotransfected into HOS (A) and 143B (B) cells, and a luciferase reporter assay was applied to detect the luciferase activities, individually. C and D, A RNA‐pull down assay was performed by biotinylated antisense oligos specific targeting of circPVT1 and FOXC2 after transfecting with oecircPVT1 or sicircPVT1 in HOS (C) and 143B (D) cells. Relative miR‐526b pulled down by circPVT1 or FOXC2 mRNA was measured by a qRT‐PCR assay. E and F, A transwell migration assay was performed in HOS cells (E) with four groups (vector, oecircPVT1, oecircPVT1‐mut and oecircPVT1+miR‐526b mimic), and a transwell invasion assay was performed in 143B cells (F) with four groups (vector, oecircPVT1, oecircPVT1‐mut and oecircPVT1+miR‐526b mimic), either. G and H, Migration ability of HOS (G) and 143B (H) cells was determined by a wound healing assay. Each sample was run in triplicate and in multiple experiments for mean ± SD. ^n.s^ *P* > .05 and **P < .01 compared to controls, respectively

Circular RNAs are reported as working as ceRNAs to regulate their downstream mRNAs via miRNAs sponging. Therefore, we wondered the ceRNA network among circPVT1, miR‐526b and FOXC2 do exist. We firstly constructed luciferase assay to elucidate the directly binding effect between circPVT1 and miR‐526b. As the findings presented in Figure 6A, B, cotransfection of miR‐526b mimic and wild‐type of reporter plasmids (circPVT1‐luc‐wt) that containing wild‐type of miR‐526b binding sites, the luminance was significantly decreased. When the theoretical binding sites of miR‐526b in circPVT1 was mutated (cotransfection of miR‐526 mimic and circPVT1‐luc‐mut), the luminance was re‐enhanced. Subsequently, we performed a RNA‐pull down assay to further conform the interaction between miR‐526b and circPVT1/FOXC2. We added circPVT1 overexpression plasmids oecircPVT1 and specific siRNAs (sicircPVT1) into HOS and 143B cells. Specific biotinylated antisense oligos were used to isolate circPVT1 and FOXC2 from the above OS cells, individually. A qRT‐PCR assay was then applied to analyse the abundance of miR‐526b that was pulled down by circPVT1 or FOXC2 mRNA. As the data showed in Figure 6C, comparing with vector, overexpression of circPVT1 resulted to an increased abundance of miR‐526b that were pulled down by circPVT1 but a decreased abundance of miR‐526b that were pulled down by FOXC2. By contrast, when circPVT1 was knocked down by sicircPVT1, the pulled‐down miR‐526b by circPVT1 was decreased but increased by FOXC2 (Figure 6D). Functionally, we performed a transwell assay and a wound healing assay to illustrate the role of circPVT1/miR‐526b/FOXC2 axial working on OC cells migration and invasion. As the representative photographs showed in Figure 6E‐H, a wild circPVT1 overexpression plasmid (oecircPVT1) who contained miR‐526b response elements (MRE‐526b) promoted OS cells migration and invasion. When the theoretical binding sites that circPVT1 provided for miR‐526b was mutated (transfection of oecircPVT1‐mut), the facilitative effect t was abolished. Additionally, we found that the promotive effect of oecircPVT1‐wt on OS cells migration and invasion was attenuated by an up‐regulation of miR‐526b (cotransfection of oecircPVT1‐wt and miR‐526b mimics).

## 4 DISCUSSION

Circular RNAs (circRNAs) are a class of endogenous non‐coding RNAs that can form between a downstream 3’ splice site and an upstream 5’ splice site in a linear precursor mRNA (pre‐mRNA).^31^ For lacking of 3’ termini and being resistant to degradation by exonuclease RNase R, circRNAs are more stable than associated linear mRNAs.^32^ CircPVT1 is located at chromosome 8q24.21 and is generated by circularization of an exon of the PVT1 pre‐lncRNA.^25^ CircPVT1 is more commonly working as an oncogene in various cancers. Zhu PK reported that circPVT1 was overexpressed in osteosarcoma and facilitated to doxorubicin and cisplatin resistance of osteosarcoma cells via regulating the expression of classical drug resistance‐related gene ATP binding cassette subfamily B member 1 (ABCB1).^33^ Li X found that circPVT1 promoted NSCLC cells proliferation and invasion via regulation of E2F transcription factor 2 (E2F2) signalling through miR‐125b sponging.^34^ In the present study, we found that circPVT1 was up‐regulated in OS tissue samples and cell lines. In addition, we showed that circPVT1 had remarkable clinical value in OS for its closed correlation with OS patients’ clinicopathological features as clinical staging, TNM staging and survival rate. A further loss of function assay presented that a knockdown of circPVT1 suppressed OS cells migration and invasion, and this phenotype test indicated that circPVT1 was involved in OS cells metastasis.

As a member of FOX proteins, FOXC2 who also termed mesenchyme forkhead 1, is known as winged‐helices owing to the butterfly‐like appearance of the loops in the protein structures.^35^ FOXC2 is composed of a single exon located on the chromosomal band 16p24.1, structurally.^36^ FOXC2 is widely accepted as an oncogene via interacting with cadherin family, protein kinases and other molecules to promote cell proliferation and metastasis in cancers.^37^ In the present study, we also concentrated on the relationship between circPVT1 and FOXC2. We found that circPVT1 positively regulated FOXC2 protein expression, and a block of FOXC2 reversed the facilitative effect of circPVT1 working on OS cells metastasis. These findings strongly indicated that FOXC2 was a key downstream metastasis‐related gene of circPVT1 in OS. A further qRT‐PCR assay demonstrated that circPVT1 had no significant effect on FOXC2 mRNA expression. This phenomenon suggested that circPVT1 regulated FOXC2 at post‐transcriptional level. It is well known that RNA transcripts communicate through the language of ceRNA which is mediated by microRNAs (miRNAs).^38^ Thereby, we hypothesis that circPVT1 might cowork with any miRNAs to regulate FOXC2.

**FIGURE 7:**
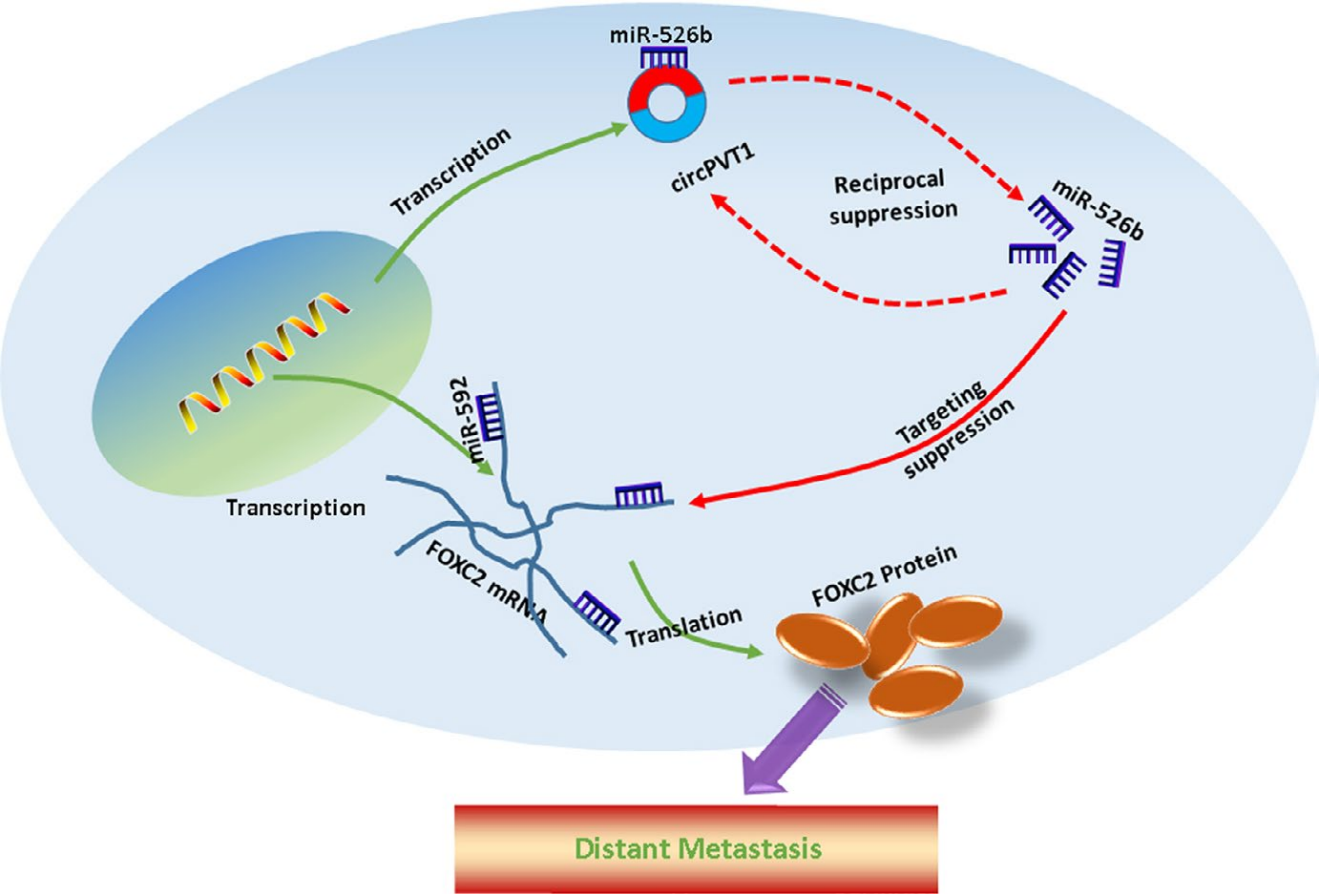
Schematic diagram of mechanism on this research. CircPVT1 promoted metastasis via regulating of miR‐526b/FOXC2 signals in OS cells.

MiRNAs, length of 20‐22 nucleotides, are a great amount of small RNAs with non‐protein coding abilities. MiRNAs negative regulate gene expression via decreasing the stability of target RNAs or limiting their translation.^39^ An online prediction draws our attention to microRNA‐526b (miR‐526) for the similar MREs it provided for both circPVT1 and FOXC2. MiR‐526b is located at human chromosome 19q13.42 and contains one exon. MiR‐526b was reported as a tumour suppressor in various cancers including cervical cancer, hepatocellular carcinoma, colon cancer and glioma.^40^, ^41^, ^42^, ^43^ In the current research, we checked the expression level of miR‐526b in OS. As expected, we showed that miR‐526b was down‐regulated in OS. We also found that miR‐526b negatively correlated with FOXC2, and up‐ and down‐regulation of miR‐526b inversely affected the regulative effect of circPVT1 on FOXC2, and these findings indicated that miR‐526b was a key molecule connecting circPVT1 and FOXC2. To functionally verify the role of miR‐526b on OS cells metastasis, we performed a transwell assay as well as a luciferase assay and found that miR‐526b suppressed OS cells metastasis via targeting of FOXC2. Taking all, as the diagram explained in Figure 7, our findings of the present research uncovered that circPVT1 promoted metastasis via regulating of miR‐526b/FOXC2 signals in OS cells.

Metastasis of OS is an intricate biological process drawing various molecules. Our findings firstly demonstrated the role of circPVT1/miR‐52b/FOXC2 axis playing in OS metastasis. Our findings might provide a novel therapeutic target in molecular treatment of OS.

## CONFLICT OF INTEREST

The authors declare no conflict of interest.

## AUTHORS' CONTRIBUTIONS

Ming Yan, Hang Gao and Wei Liu conceived the experiments; Zhenshan Lv, Ying Liu and Song Zhao performed the experiments; Weiquan Gong and Wei Liu analysed the data; Wei Liu wrote the manuscript. All authors read and approved the final manuscript.

## Supporting information

Figure S1

Figure S2

Table S1

## References

[1] Philip T , Blay JY , Brunat‐Mentigny M , et al. Osteosarcoma. Br J Cancer. 2001;84(Suppl 2):78‐80. 11355976 10.1054/bjoc.2001.1770 PMC2408847

[2] Meazza C , Scanagatta P . Metastatic osteosarcoma: a challenging multidisciplinary treatment. Expert Rev Anticancer Ther. 2016;16:543‐556. 26999418 10.1586/14737140.2016.1168697

[3] Wang C , Ren M , Zhao X , Wang A , Wang J . Emerging roles of circular RNAs in osteosarcoma. Med Sci Monit. 2018;24:7043‐7050. 30282962 10.12659/MSM.912092 PMC6183101

[4] Jeck WR , Sharpless NE . Detecting and characterizing circular RNAs. Nat Biotechnol. 2014;32:453‐461. 24811520 10.1038/nbt.2890 PMC4121655

[5] Rybak‐Wolf A , Stottmeister C , Glazar P , et al. Circular RNAs in the mammalian brain are highly abundant, conserved, and dynamically expressed. Mol Cell. 2015;58:870‐885. 25921068 10.1016/j.molcel.2015.03.027

[6] Han D , Li J , Wang H , et al. Circular RNA circMTO1 acts as the sponge of microRNA‐9 to suppress hepatocellular carcinoma progression. Hepatology. 2017;66:1151‐1164. 28520103 10.1002/hep.29270

[7] Su H , Tao T , Yang Z , et al. Circular RNA cTFRC acts as the sponge of MicroRNA‐107 to promote bladder carcinoma progression. Mol Cancer. 2019;18:27. 30782157 10.1186/s12943-019-0951-0 PMC6379985

[8] Wu K , Liao X , Gong Y , et al. Circular RNA F‐circSR derived from SLC34A2‐ROS1 fusion gene promotes cell migration in non‐small cell lung cancer. Mol Cancer. 2019;18:98. 31118036 10.1186/s12943-019-1028-9 PMC6530145

[9] Wu Y , Xie Z , Chen J , et al. Circular RNA circTADA2A promotes osteosarcoma progression and metastasis by sponging miR‐203a‐3p and regulating CREB3 expression. Mol Cancer. 2019;18:73. 30940151 10.1186/s12943-019-1007-1 PMC6444890

[10] Xia L , Song M , Sun M , Wang F , Yang C . Circular RNAs as Biomarkers for Cancer. Adv Exp Med Biol. 2018;1087:171‐187. 30259366 10.1007/978-981-13-1426-1_14

[11] Xu XW , Zheng BA , Hu ZM , et al. Circular RNA hsa_circ_000984 promotes colon cancer growth and metastasis by sponging miR‐106b. Oncotarget. 2017;8:91674‐91683. 29207676 10.18632/oncotarget.21748 PMC5710956

[12] Zhang J , Liu H , Hou L , et al. Circular RNA_LARP4 inhibits cell proliferation and invasion of gastric cancer by sponging miR‐424‐5p and regulating LATS1 expression. Mol Cancer. 2017;16:151. 28893265 10.1186/s12943-017-0719-3 PMC5594516

[13] Verduci L , Ferraiuolo M , Sacconi A , et al. The oncogenic role of circPVT1 in head and neck squamous cell carcinoma is mediated through the mutant p53/YAP/TEAD transcription‐competent complex. Genome Biol. 2017;18:237. 29262850 10.1186/s13059-017-1368-y PMC5738800

[14] Memczak S , Jens M , Elefsinioti A , et al. Circular RNAs are a large class of animal RNAs with regulatory potency. Nature. 2013;495:333‐338. 23446348 10.1038/nature11928

[15] Qin S , Zhao Y , Lim G , Lin H , Zhang X , Zhang X . Circular RNA PVT1 acts as a competing endogenous RNA for miR‐497 in promoting non‐small cell lung cancer progression. Biomed Pharmacother. 2019;111:244‐250. 30590312 10.1016/j.biopha.2018.12.007

[16] Wang Z , Su M , Xiang B , Zhao K , Qin B . Circular RNA PVT1 promotes metastasis via miR‐145 sponging in CRC. Biochem Biophys Res Comm. 2019;512:716‐722. 30922567 10.1016/j.bbrc.2019.03.121

[17] Cai J , Tian AX , Wang QS , et al. FOXF2 suppresses the FOXC2‐mediated epithelial‐mesenchymal transition and multidrug resistance of basal‐like breast cancer. Cancer Lett. 2015;367:129‐137. 26210254 10.1016/j.canlet.2015.07.001

[18] Zhang CL , Zhu KP , Ma XL . Antisense lncRNA FOXC2‐AS1 promotes doxorubicin resistance in osteosarcoma by increasing the expression of FOXC2. Cancer Lett. 2017;396:66‐75. 28323030 10.1016/j.canlet.2017.03.018

[19] Zheng CH , Quan Y , Li YY , Deng WG , Shao WJ , Fu Y . Expression of transcription factor FOXC2 in cervical cancer and effects of silencing on cervical cancer cell proliferation. Asian Pac J Cancer Prev. 2014;15:1589‐1595. 24641373 10.7314/apjcp.2014.15.4.1589

[20] Zhu JL , Song YX , Wang ZN , et al. The clinical significance of mesenchyme forkhead 1 (FoxC2) in gastric carcinoma. Histopathology. 2013;62:1038‐1048. 23614500 10.1111/his.12132

[21] Cui YM , Jiao HL , Ye YP , et al. FOXC2 promotes colorectal cancer metastasis by directly targeting MET. Oncogene. 2015;34:4379‐4390. 25381815 10.1038/onc.2014.368

[22] Gozo MC , Jia D , Aspuria PJ , et al. FOXC2 augments tumor propagation and metastasis in osteosarcoma. Oncotarget. 2016;7:68792‐68802. 27634875 10.18632/oncotarget.11990 PMC5356590

[23] Zhu KP , Zhang CL , Shen GQ , Zhu ZS . Long noncoding RNA expression profiles of the doxorubicin‐resistant human osteosarcoma cell line MG63/DXR and its parental cell line MG63 as ascertained by microarray analysis. Int J Clin Exp Pathol. 2015;8:8754‐8773. 26464619 PMC4583851

[24] Chen J , Li Y , Zheng Q , et al. Circular RNA profile identifies circPVT1 as a proliferative factor and prognostic marker in gastric cancer. Cancer Lett. 2017;388:208‐219. 27986464 10.1016/j.canlet.2016.12.006

[25] Panda AC , Grammatikakis I , Kim KM , et al. Identification of senescence‐associated circular RNAs (SAC‐RNAs) reveals senescence suppressor CircPVT1. Nucleic Acids Res. 2017;45:4021‐4035. 27928058 10.1093/nar/gkw1201 PMC5397146

[26] Wang Y , Lu Z , Wang N , et al. Long noncoding RNA DANCR promotes colorectal cancer proliferation and metastasis via miR‐577 sponging. Exp Mol Med. 2018;50:57. 10.1038/s12276-018-0082-5 PMC5938019 29717105

[27] Wang K , Jin W , Song Y , Fei X . LncRNA RP11‐436H11.5, functioning as a competitive endogenous RNA, upregulates BCL‐W expression by sponging miR‐335‐5p and promotes proliferation and invasion in renal cell carcinoma. Mol Cancer. 2017;16:166. 29070041 10.1186/s12943-017-0735-3 PMC5657097

[28] Kume T . The role of FoxC2 transcription factor in tumor angiogenesis. J Oncol. 2012;2012:204593. 22174714 10.1155/2012/204593 PMC3228356

[29] Werden SJ , Sphyris N , Sarkar TR , et al. Phosphorylation of serine 367 of FOXC2 by p38 regulates ZEB1 and breast cancer metastasis, without impacting primary tumor growth. Oncogene. 2016;35:5977‐5988. 27292262 10.1038/onc.2016.203 PMC5114155

[30] Dudekula DB , Panda AC , Grammatikakis I , De S , Abdelmohsen K , Gorospe M . CircInteractome: A web tool for exploring circular RNAs and their interacting proteins and microRNAs. RNA Biol. 2016;13:34‐42. 26669964 10.1080/15476286.2015.1128065 PMC4829301

[31] Chen LL . The biogenesis and emerging roles of circular RNAs. Nat Rev Mol Cell Biol. 2016;17:205‐211. 26908011 10.1038/nrm.2015.32

[32] Chuang TJ , Wu CS , Chen CY , Hung LY , Chiang TW , Yang MY . NCLscan: accurate identification of non‐co‐linear transcripts (fusion, trans‐splicing and circular RNA) with a good balance between sensitivity and precision. Nucleic Acids Res. 2016;44:e29. 26442529 10.1093/nar/gkv1013 PMC4756807

[33] Kun‐Peng Z , Xiao‐Long M , Chun‐Lin Z . Overexpressed circPVT1, a potential new circular RNA biomarker, contributes to doxorubicin and cisplatin resistance of osteosarcoma cells by regulating ABCB1. Int J Biol Sci. 2018;14:321‐330. 29559849 10.7150/ijbs.24360 PMC5859477

[34] Li X , Zhang Z , Jiang H , et al. Circular RNA circPVT1 promotes proliferation and invasion through sponging miR‐125b and activating E2F2 signaling in non‐small cell lung cancer. Cell Physiol Biochem. 2018;51:2324‐2340. 30537738 10.1159/000495876

[35] Arden KC . Multiple roles of FOXO transcription factors in mammalian cells point to multiple roles in cancer. Exp Gerontol. 2006;41:709‐717. 16806782 10.1016/j.exger.2006.05.015

[36] Miura N , Wanaka A , Tohyama M , Tanaka K . MFH‐1, a new member of the fork head domain family, is expressed in developing mesenchyme. FEBS Lett. 1993;326:171‐176. 8325367 10.1016/0014-5793(93)81785-x

[37] Wang T , Zheng L , Wang Q , Hu YW . Emerging roles and mechanisms of FOXC2 in cancer. Clin Chim Acta. 2018;479:84‐93. 29341903 10.1016/j.cca.2018.01.019

[38] Salmena L , Poliseno L , Tay Y , Kats L , Pandolfi PP . A ceRNA hypothesis: The Rosetta stone of a hidden RNA language? Cell. 2011;146(3):353‐358. 21802130 10.1016/j.cell.2011.07.014 PMC3235919

[39] Fabian MR , Sonenberg N , Filipowicz W . Regulation of mRNA translation and stability by microRNAs. Annu Rev Biochem. 2010;79:351‐379. 20533884 10.1146/annurev-biochem-060308-103103

[40] Li H , Wang J , Xu F , et al. By downregulating PBX3, miR‐526b suppresses the epithelial‐mesenchymal transition process in cervical cancer cells. Future Oncol. 2019;15:1577‐1591. 30859853 10.2217/fon-2018-0575

[41] Liu X , Yang L , Tu J , et al. microRNA‐526b servers as a prognostic factor and exhibits tumor suppressive property by targeting Sirtuin 7 in hepatocellular carcinoma. Oncotarget. 2017;8:87737‐87749. 29152116 10.18632/oncotarget.21209 PMC5675668

[42] Wu M , Li X , Liu Q , Xie Y , Yuan J , Wanggou S . miR‐526b‐3p serves as a prognostic factor and regulates the proliferation, invasion, and migration of glioma through targeting WEE1. Cancer Manag Res. 2019;11:3099‐3110. 31114353 10.2147/CMAR.S192361 PMC6489667

[43] Zhang R , Zhao J , Xu J , Wang J , Jia J . miR‐526b‐3p functions as a tumor suppressor in colon cancer by regulating HIF‐1alpha. Am J Transl Res. 2016;8:2783‐2789. 27398161 PMC4931172

