# Supplementary figures and images for: Circular RNA PVT1 promotes metastasis via regulating of miR‐526b/FOXC2 signals in OS cells
Source: J Cell Mol Med. 2020 Apr 5;24(10):5593–604. doi: 10.1111/jcmm.15215 (PMC7214167; doi:10.1111/jcmm.15215)

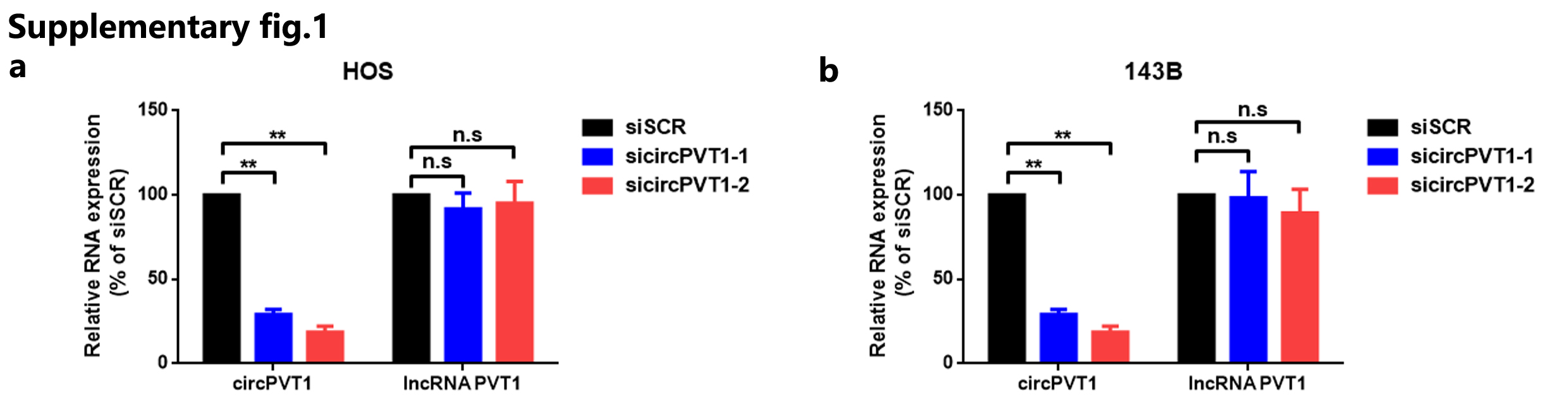

Supplement: Supplementary file 1 — Figure S1 [file JCMM-24-5593-s001.jpg]

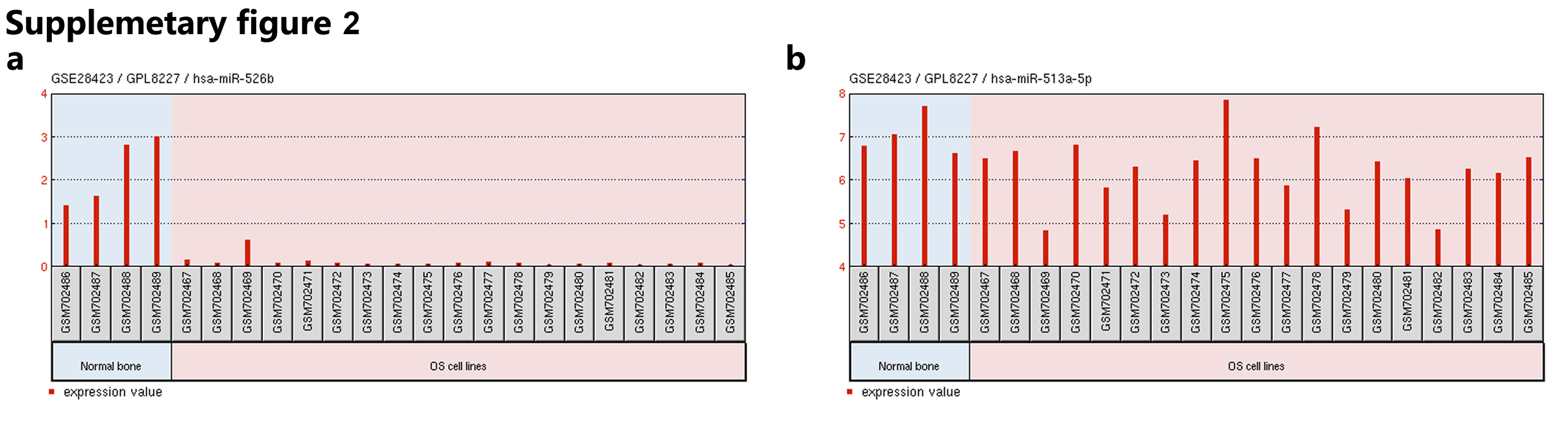

Supplement: Supplementary file 2 — Figure S2 [file JCMM-24-5593-s002.jpg]
