# Supplementary material for: Circular RNA PVT1 promotes metastasis via regulating of miR‐526b/FOXC2 signals in OS cells
Source: J Cell Mol Med. 2020 Apr 5;24(10):5593–604. doi: 10.1111/jcmm.15215 (PMC7214167; doi:10.1111/jcmm.15215)
Supplement: Supplementary file 3 — Table S1 [file JCMM-24-5593-s003.docx]

| Gene | Sequences of primers/oligonucleotides |
| --- | --- |
| circPVT1 forward | CGACTCTTCCTGGTGAAGCATCTGAT |
| circPVT1 reverse | TACTTGAACGAAGCTCCATGCAGC |
| FOXC2 forward | GCCCAGCAGCAAACTTTCC |
| FOXC2 reverse | CCGGTGGGAGTTGAACATCT |
| GAPDH forward | TGTTCGTCATGGGTGTGAAC |
| GAPDH reverse | ATGGCATGGACTGTGGTCAT |
| miR-526b forward | GTCTCTTGAGGGAAGCACT |
| miR-526b reverse | GTGCAGGGTCCGAGGT |
| U6 forward | CGCTTCGGCAGCACATATACTA |
| U6 reverse | CGCTTCACGAATTTGCGTGTCA |
| sicircPVT1-1 | GCAAAUGAAAGCUACCAAUTT |
| sicircPVT1-2 | GCACAAUAUCUUUGAACUATT |
| miR-526b mimics | CTCTTGAGGGAAGCACTTTCTGT |
| miR-526b inhibitor | ACAGAAAGTGCTTCCCTCAAGAG |

**Supplementary Table 1. Primer sequences and oligonucleotides used in this research**
